# Army Legislation

**Published:** 1900-03

**Authors:** 


### ARMY LEGISLATION.

The fate of army legislation is now in the balance, the outposts have all been won, and the lines of action are now clear for the final charge. The Army Department, from the Secretary of War to the Adjutant-General and his staff, must be won to our cause to make victory sure. Every influence in our power should be brought upon Secretary Root and his staff to give their unqualified support to this long-deferred measure. There are many avenues to Washington, and hurried tread should be the command to every supporter of this measure, whose ways can lead to the War Department. Let everyone respond to the final call and send forth a final shot to win this victory.

